# Anti proliferative activity of ELACYT™ (CP-4055) in combination with cloretazine (VNP40101M), idarubicin, gemcitabine, irinotecan and topotecan in human leukemia and lymphoma cells

**DOI:** 10.1080/10428190801935752

**Published:** 2008-04-08

**Authors:** David J. Adams, Marit L. Sandvold, Finn Myhren, Tove F. Jacobsen, Frank Giles, David A. Rizzieri

**Affiliations:** ^1^Department of Medicine, Duke University Medical Center, Durham, NC, USA; ^2^Preclinical, Chemical and Pharmaceutical Research and Development, Clavis Pharma ASA, NO-0256 Oslo, Norway; ^3^University of Texas Health Science Center, San Antonio, TX, USA

**Keywords:** DNA antimetabolite, combination chemotherapy, drug synergy, median effect analysis, leukemia, lymphoma

## Abstract

This study evaluated combination drug partners for CP-4055, the C18:1^Δ9,trans^ unsaturated fatty acid ester of cytarabine in HL-60 and U937 cells. Growth inhibition was assessed by ATP assay and drug interaction by the combination index and three dimensional methods. Synergy was observed in HL-60 cells for simultaneous combinations of CP-4055 with gemcitabine, irinotecan and topotecan, while combinations with cloretazine (VNP40101M) and idarubicin were additive. In U937 cells, synergy was observed with gemcitabine and additivity for the other drugs. In HL-60, the IC50 concentration of CP-4055 could be reduced 10-fold and that of gemcitabine 3-fold in combination versus the agents alone, an interaction that was independent of drug sequence, ratio and exposure time. In contrast, interactions of CP-4055 with the topoisomerase inhibitors became antagonistic when the drugs were administered 24 h prior to CP-4055 and at certain drug ratios, particularly in U937 cells. In summary, CP-4055 produced additive to synergistic anti proliferative activity when combined simultaneously with drugs from four mechanistic classes in cell culture models of human leukemia and lymphoma. The impact of drug sequence and ratio on the interactions argues for incorporation of these parameters into the design of combination chemotherapy regimens.

## Introduction

Cytarabine (ara-C) is the most active single agent in acute myelogenous leukemia (AML) and is also important for treatment of acute lymphocytic leukemia, chronic myelogenous leukemia and non-Hodgkin lymphoma. The active metabolite of cytarabine is ara-CTP and monophosphorylation by deoxycytidine kinase (dCK) is the rate-limiting step in its synthesis. Ara-CTP causes both reversible and irreversible inhibition of DNA synthesis via inhibition of DNA polymerase and by incorporation into DNA with induction of apoptosis, respectively [1]. Accumulation of ara-CTP in leukemic blasts correlates with clinical response; however, this process is limited by rapid deamination to uracil arabinoside by cytidine deaminase in blood, liver, kidney and intestine. Cellular resistance to cytarabine can also occur via multiple mechanisms, including altered nucleoside transport and reduced intracellular dCK activity [2,3]. Combination chemotherapy is often employed to overcome such limitations and synergistic interactions of cytarabine with purine nucleoside analogs have been exploited for some time [4]. Recently, synergy has also been reported for combinations of cytarabine with gemcitabine, another pyrimidine nucleoside analog [5 – 7].

To enhance cellular uptake, decrease inactivation and prolong exposure to ara-CTP, the elaidic acid ester of cytarabine, CP-4055 was developed. Accordingly, CP-4055 enters cells by a nucleoside transporter-independent mechanism and exhibits longer intracellular retention with accompanying longer inhibition of DNA synthesis [8 – 10]. Unlike ara-C, CP-4055 also causes transient inhibition of RNA synthesis, is active in cytarabine-resistant tumours and has Phase I activity in solid tumours, including melanoma, non-small cell lung cancer and lung cancer refractory to gemcitabine [11,12]. Clinically, CP-4055 is well tolerated in patients with hematologic malignancies at bolus doses of 875 mg/m^2^/d and by continuous infusion of 675 mg/m^2^/d given daily for 5 days every 3 weeks [13]. The present study was undertaken to identify combination drug partners for CP-4055 in acute myeloid leukemia and lymphoma. Two complementary approaches were utilised to evaluate drug interaction: the combination index and combination effect methods. In addition, the study sought to define optimal treatment parameters for synergistic or additive drug combinations, including effects of drug sequencing, drug ratio and the relative impact of drug concentration and time of exposure on tumor growth inhibition. The results underscore the importance of these parameters in drug interaction, since the type of interaction was highly sequence and ratio-dependent for certain combinations. Overall, the synergistic anti proliferative activity of CP-4055 combined with gemcitabine proved to be the most robust, being largely independent of sequence, ratio and exposure time in both leukemia and lymphoma cell models.

## Methods

### Drugs and chemicals

CP-4055 was obtained from Clavis ASA (Oslo, Norway) and stored at room temperature. Cloretazine™ (VNP40101M; Vion Pharmaceuticals, Inc. New Haven, CT), and idarubicin (Sigma-Aldrich, St. Louis, MO) were stored at 2 – 8°C. Concentrated stock solutions of these agents were prepared in cell culture grade dimethylsulfoxide (Sigma-Aldrich) and stored at −20°C. Clinical formulations of cytarabine, gemcitabine, topotecan and irinotecan were obtained from Bedford Laboratories (Bedford, MA), Eli Lilly (Indianapolis, IN), Glaxo SmithKline (Research Triangle Park, NC), and Pfizer (New London, CT), respectively and stored at room temperature.

### Cell lines and culture conditions

Cell lines were obtained from the American Type Culture Collection, Manassas, VA through the Duke Cell Culture Facility and were mycoplasma-free. HL-60 is a promyelocytic cell line derived from peripheral blood leukocytes of a 36-year-old Caucasian female with acute promyelocytic leukemia [14]. The cells are pseudo-diploid, p53 negative and express the myc oncogene, but do not express the PML-RAR fusion protein commonly found in APL. The cells also have a deletion of the 5q31 region, a frequent clonal chromosomal abnormality found in human myelodysplastic syndrome and acute myeloid leukemia that is thought to contribute to the pathogenesis of these diseases by deleting one or more tumor-suppressor genes [15]. HL-60 cells were cultured in Iscove's-modified Dulbecco's medium (ATCC, Manassas, VA), containing 4 mM l-glutamine, 1.5 g/L sodium bicarbonate and 20% fetal bovine serum (HyClone, Logan, UT). The U937 cell line was derived from the pleural effusion of a 37-year-old male Caucasian with histiocytic lymphoma [16]. The cells were cultured in RPMI 1640 medium containing 4.5 g/L glucose, 10 mM HEPES, 1 mM pyruvate and 10% fetal bovine serum. Both cell lines were maintained in log phase growth at a density of 4 × 10^5^ −1 × 10^6^ cells/mL.

### Growth inhibition and drug interaction assays

Growth inhibition and drug interaction in vitro assays have been described previously [17]. Briefly, cells were seeded at 2 × 10^4^/well/200 μL in 96 well conical-bottom microplates. Following drug addition one (HL-60) or 24 h (U937) later, cells were incubated for 48 h (∼2 cell doublings) and then 40 μL/well were assayed for ATP as a biomarker for viable surviving cells utilizing the ATPLite™-M luminescence Assay System (Perkin-Elmer, Waltham, MA). The relation of cell number to luminescence was linear from 2 × 10^5^ to 10 cells/well. Mean fractional growth inhibition, defined as (treated – blank)/(untreated control – blank) was determined from 3 – 4 replicate wells per dose. Individual dose-response curves were then constructed and the IC50 computed from the best fitting transition functions (determined by F-statistic) using TableCurve 2D curve-fitting software (SPSS, Downers Grove, IL). The IC50 is defined as the drug concentration producing 50% growth inhibition relative to untreated controls. Alternatively, composite dose response curves were obtained from multiple experiments and the median effective dose, *D*_m_ (equivalent to the IC50), computed using CalcuSyn software (Biosoft, Cambridge, UK; an updated version of this software, termed CompuSyn is now available from ComboSyn, Inc., Paramus, NJ).

Drug interaction was assessed by the combination index method of Chou and Talalay (originally described in [18]; recently reviewed in [19]). This method is based on the median effect principle:

(1) $$f_{\text{a}}/f_{\text{u}}{\left(D/D_{\text{m}}\right)}^{\text{m}}$$

where *D* is administered dose, *D*_m_ is the dose that yields 50% growth inhibition, *f*_a_ is the cell fraction affected by dose *D*, *f*_u_ is the unaffected fraction, and *m* is a coefficient that defines the sigmoidicity of the dose-effect curve.

This relationship and the law of mass action lead to a generalised equation for the interaction of multiple inhibitors

(2) $${\left(f_{\text{a}}\right)}_{\text{A},\text{B}}/{\left(f_{\text{u}}\right)}_{\text{A},\text{B}}={\left(f_{\text{a}}\right)}_{\text{A}}/{\left(f_{\text{u}}\right)}_{\text{B}}+{\left(f_{\text{a}}\right)}_{\text{B}}/{\left(f_{\text{u}}\right)}_{\text{B}}+\alpha {\left(f_{\text{a}}\right)}_{\text{A}}{\left(f_{\text{a}}\right)}_{\text{B}}/{\left(f_{\text{u}}\right)}_{\text{A}}{\left(f_{\text{u}}\right)}_{\text{B}}$$

where (*f*_a_)_A_, (*f*_u_)_B_ and (*f*_a_)_A,B_ are the fractions affected by drugs A and B alone and in combination, respectively. From Eqs. (1) and (2), the equation for the combination index (CI) can be derived as

(3) $$\text{CI}={\left(D\right)}_{\text{A}}/{\left(D_{\text{x}}\right)}_{\text{A}}+{\left(D\right)}_{\text{B}}/{\left(D_{\text{x}}\right)}_{\text{B}}+\alpha {\left(D\right)}_{\text{A}}{\left(D\right)}_{\text{B}}/{\left(D_{\text{x}}\right)}_{\text{A}}{\left(D_{\text{x}}\right)}_{\text{B}}$$

Where D is the dose that yields x% growth inhibition and α = 0 for mutually exclusive drugs and α = 1 for mutually non-exclusive drugs. Additivity is then defined as CI = 1; synergy as CI < 1; and antagonism as CI > 1.

This study used the mutually exclusive assumption (the drugs have similar sites of action) as incorporated in CalcuSyn software from at least three replicate experiments.

Drug interaction was also assessed by the combination effect method of Kanzawa [20], which is based on the three-dimensional model of Pritchard and Shipman [21], and considers the drugs mutually-non-exclusive.

This method defines theoretical additivity (TA) based on Eq. (2) above as

(4) $${\text{TA}}_{\left(\text{t}\right)}={\left(f_{\text{a}}\right)}_{\text{A}}+{\left(f_{\text{a}}\right)}_{\text{B}}-{\left(f_{\text{a}}\right)}_{\text{A}}{\left(f_{\text{a}}\right)}_{\text{B}}$$

The combination effect (CE) surface is then given by

(5) $${\left\{{\left(\text{S}a\right)}_{\text{A},\text{B}}\right\}}_{\text{CE}}={\left\{{\left(\text{S}a\right)}_{\text{A},\text{B}}\right\}}_{\text{obs}}-{\left\{{\left(\text{S}a\right)}_{\text{A},\text{B}}\right\}}_{\text{cal}}$$

Here {(S*a*)_A,B_}_obs_ represents the observed effect, and {(S*a*)_A,B_}_cal_ is the theoretical response surface, given by

(6) $${\left\{{\left(\text{S}a\right)}_{\text{A},\text{B}}\right\}}_{\text{cal}}=\int_{a=o}^{n}\int_{b=0}^{m}\left({\left(f_{a}\right)}_{\text{A}}+{\left(f_{a}\right)}_{\text{B}}-{\left(f_{a}\right)}_{\text{A}}{\left(f_{a}\right)}_{\text{B}}\right)$$

Additivity is defined as CE = 0; synergy as CE < 0; and antagonism as CE > 0.

This complementary approach was performed in 8 × 8 checkerboard format in six replicate plates. The associated combination effect surface was computed from mean values normalised by the respective 95% confidence limit. For sequencing studies, drug exposures were separated by 24 h.

For studies addressing the impact of exposure time on anti proliferative activity, replicate dose-response plates were prepared that included CP-4055 alone, gemcitabine alone, and CP-4055 combined with gemcitabine at a ratio of 1:0.0025 (HL-60) or 1:0.02 (U937). Following drug exposure times of 1, 3, 9, 27 and 54 h, plates were centrifuged for 5 min on a Sorvall RT-7 centrifuge at 1000 rpm. Culture medium was removed and replaced with fresh drug-free medium for a minimum of 3 h. Cells were then assayed for ATP as described above.

## Results

### Rationale for the choice of combination partners

**Figure 1:**
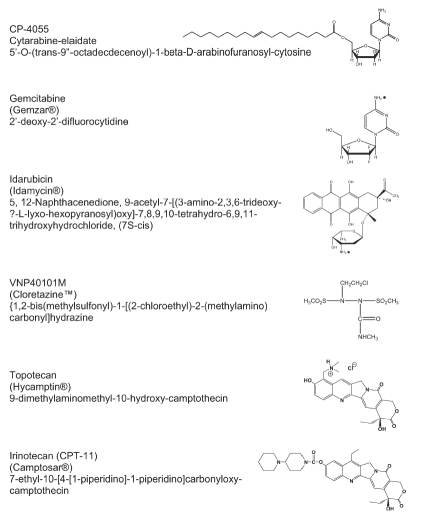
Structures of drugs under study.

The choice of potential combination partners for CP-4055 included drugs with diverse structures (Figure 1) and mechanisms of action that have documented clinical activity in leukemia/lymphoma. For example, many chemotherapy regimens for AML contain an anthracycline, which causes DNA damage via inhibition of topoisomerase II. Idarubicin represents a second generation anthracycline with clinical activity in AML that is less cardiotoxic and less susceptible to multi-drug resistance. Similarly, topotecan and irinotecan are topoisomerase I inhibitors clinically approved for use in ovarian and small cell lung cancer and in colorectal cancer, respectively. Irinotecan is active in combination with cytarabine in refractory AML [22], while topotecan is activ alone and in combination in hematological malignancies [23]. Gemcitabine is a nucleoside analog with activity in both hematological and solid tumours. As a DNA antimetabolite, gemcitabine causes masked chain termination during DNA synthesis, which is difficult to repair. In addition, the dFdCDP metabolite of gemcitabine potentiates the drug’s cytotoxicity. dFdCDP inhibits ribonucleotide reductase, which depletes deoxynucleotide pools that would otherwise inhibit deoxycytidine kinase, the rate-limiting enzyme in formation of dFdCMP from gemcitabine as well as ara-CMP from cytarabine. Gemcitabine can also act by inhibiting topoisomerase I [24]. Cloretazine is a new antitumor agent that has shown clinical activity in relapsed or refractory AML [25]. Cloretazine is a pro-drug whose unique activation produces both carbamoylating and alkylating species that lead to DNA damage [26].

### CP-4055 exhibits significant anti proliferative activity in HL-60 and U937 cells

**Figure 2:**
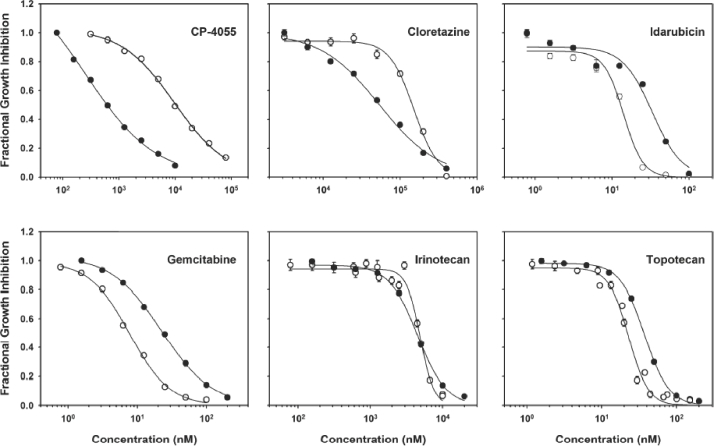
Composite dose-response curves in human leukemia cells. Data from replicate dose-response experiments for HL-60 (open circles) and U937 (filled circles) leukemia cell lines were combined and the mean values plotted for the various drugs under study. Line: data fit to the Hill Equation (a form of the median-effect equation); bar: intra-assay standard error.

Using ATP as a metabolic endpoint, complete sigmoid dose-response curves were obtained in which the fraction of tumor cells affected ranged from 100% to ≤10% (Figure 2). While the traditional IC50 was chosen as the endpoint for comparison, an IC90 level of activity was also achieved by all seven drugs. On a molar basis, gemcitabine was the most potent agent in both cell lines, followed closely by the topoisomerase inhibitors idarubicin and topotecan (Table I). CP-4055 was three orders of magnitude less potent than gemcitabine in HL-60 and two logs less potent in U937. This is not surprising, since CP-4055, like cloretazine and irinotecan, is a pro-drug that requires enzymatic conversion by esterases and kinases to its active form, ara-CTP. Of interest, CP-4055 like cytarabine was nearly 20-fold more potent in U937 compared with HL-60. The other agents had comparable activity in each cell line, with the exception of cloretazine, which was 2-fold more active in U937 cells.

**Table I:** Potency of CP-4055 and combination partners in human leukemia cells.

	HL-60		U937	
Drug	IC50 (nM)	*N*	IC50 (nM)	*N*
CP-4055	13,387 ± 1428	24	710 ± 78	14
Cloretazine	136,150 ± 11,146	7	59,996 ± 13,852	7
Idarubicin	12.1 ± 0.6	8	30 ± 5	7
Gemcitabine	9.4 ± 0.7	18	23 ± 2	10
Irinotecan	4742 ± 253	8	3823 ± 518	7
Topotecan	20.2 ± 1.0	7	34 ± 2	7
Cytarabine	2989 ± 322	5	167 ± 29	4

### Simultaneous combinations with CP-4055 produce additive to synergistic interactions

**Figure 3:**
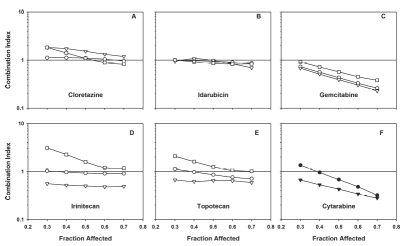
Combination index plots of Chou-Talalay and impact of drug sequence in HL-60 cells. Composite dose-response curves were constructed for drugs alone and in combination as described in Methods. The respective fraction affected (*F*_a_) was interpolated at 10 percent Intervals and the values used to compute the combination index for simultaneous administration of CP-4055 (open circles), for CP-4055 given 24 h prior to (open triangles) or 24 h after (open squares) the second drug in the combination listed in each panel. For cytarabine, only simultaneous combinations are shown combined with CP-4055 (closed circles), and with gemcitabine (closed triangles).

**Figure 4:**
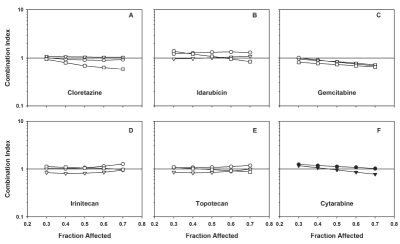
Combination index plots of Chou-Talalay and impact of drug sequence in U937 cells. Drug interaction was assessed by the combination index method as described in Figure 3 in U937 cells for the drug combination partners listed in each panel.

Median effect analysis of drugs combined simultaneously with CP-4055 at the approximate ratio of their IC50's revealed significant synergy with gemcitabine (CI = 0.4); moderate synergy with topotecan and irinotecan (CI = 0.8), and additive interactions with idarubicin and cloretazine in HL-60 cells (CI = 1.0; Table II). Addition of cloretazine or idarubicin to the CP-4055-gemcitabine combination did not significantly alter the interaction. Similar results for binary combinations were observed in U937 cells, but the synergy between CP-4055 and gemcitabine was only moderate (CI = 0.8; Table II). Important for clinical translation, no antagonistic interactions were observed with CP-4055 combinations in this setting. In contrast, combinations of idarubicin with cloretazine and with gemcitabine elicited moderately antagonistic to antagonistic responses, respectively, in HL-60 cells (Table II). Figures 3 and 4 extend the results in Table II to include interaction at various levels of effect. Of note, the CP-4055-gemcitabine combination becomes more synergistic as anti proliferative activity increases (C panels). The combination of cytarabine + gemcitabine showed synergy comparable to CP-4055 + gemcitabine in both cell lines (Figures 3 and 4, panel E).

**Table II:** Combination index at the IC50 in human leukemia cells.

	Molar ratio	*N*	CI
**HL-60 Combination**			
Cytarabine + Gemcitabine	1:0.01	3	0.39 ± 0.01
CP-4055 + Gemcitabine	1:0.0025	11	0.41 ± 0.01
CP-4055 + Topotecan	1:0.00125	6	0.78 ± 0.01
CP-4055 + Irinotecan	1:0.125	6	0.81 ± 0.07
CP-4055 + Idarubicin	1:0.0025	6	0.88 ± 0.09
CP-4055 + Cloretazine	1:10	6	0.91 ± 0.08
Cloretazine + Gemcitabine	1:0.00025	3	1.10 ± 0.11
Cloretazine + Idarubicin	1:0.00025	3	1.20 ± 0.03
Idarubicin + Gemcitabine	1:1	3	1.51 ± 0.04
CP-4055 + Cloretazine + Gemcitabine	1:10:0.001	2	0.36 ± 0.07
CP-4055 + Idarubicin + Gemcitabine	1:0.001:0.001	2	0.30 ± 0.17
CP-4055 + Cloretazine + Idarubicin	1:10:0.001	2	0.92 ± 0.15
**U937 combination**			
CP-4055 + Gemcitabine	1:0.02	8	0.82 ± 0.04
CP-4055 + Cloretazine	1:40	6	0.94 ± 0.10
Cytarabine + Gemcitabine	1:0.2	3	1.00 ± 0.05
CP-4055 + Irinotecan	1:2	4	1.11 ± 0.02
CP-4055 + Topotecan	1:0.02	4	1.11 ± 0.06
CP-4055 + Idarubicin	1:0.01	6	1.16 ± 0.11

To express the synergy of CP-4055 and gemcitabine in a more practical way, the dose reduction index was computed across various levels of affect for this combination in HL-60 cells (Table III). The results indicate that in combination, the doses of both CP-4055 and gemcitabine can be reduced significantly and yet produce the same antitumor activity as each agent alone. For example, at the IC50 (*F*_a_ = 0.5), the dose of CP-4055 can be reduced nearly 10-fold and that of gemcitabine 3-fold when used in combination. Providing the drug interaction is tumour-selective, this result suggests that reduced doses of CP-4055 combined with a relatively small amount of gemcitabine could decrease toxicity without loss of efficacy.

**Table III:** Dose reduction index for CP-4055 combined with gemcitabine in HL-60 cells.

	Dose reduction index^1^	
Fraction affected	CP-4055	Gemcitabine
0.1	2.2	1.4
0.2	3.3	1.6
0.3	4.9	1.9
0.4	6.9	2.4
0.5	9.8	3.0
0.6	14.2	3.8
0.7	21.5	4.7
0.8	35.7	5.8
0.9	71.8	6.6

1 Combined in a molar ratio of 400:1 (CP-4055:Gemcitabine).

### Drug sequence impacts drug interaction

The impact of sequence of administration on drug interaction in each cell line is shown in Figures 3 (HL-60) and 4 (U937). Results differed in the two models. In HL-60 cells, interaction of CP-4055 with the topoisomerase inhibitors exhibited a marked sequence dependence with synergy occurring when CP-4055 was given first, additivity when given together, and antagonism when CP-4055 was given second (Figure 3, panels D, E). Synergy with gemcitabine was most pronounced when the drugs were given simultaneously or when CP-4055 was administered first (panel C). The interaction of CP-4055 with cloretazine was additive except when CP-4055 was dosed first, which produced moderate antagonism (panel A). Only the additive interaction of CP-4055 with idarubicin was sequence-independent (panel B). By comparison, drug interactions in U937 cells were generally less sequence dependent, with the exception of cloretazine where synergy was observed when this agent was administered prior to CP-4055 (Figure 4, panel A).

Notably, the significant synergy seen with the topoisomerase inhibitors when preceded by CP-4055 in HL-60 cells was less pronounced in the U937 model (panels D, E).

### Drug ratio impacts anti proliferative response

**Figure 5:**
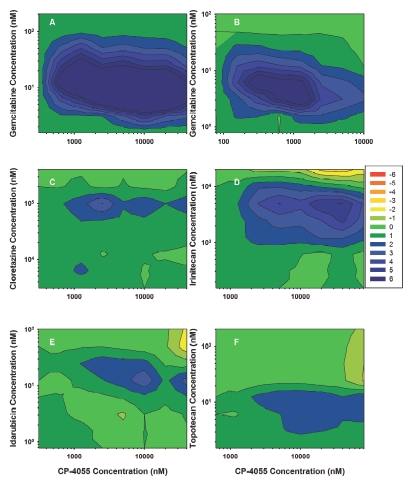
Effect of ratio on drug interaction in HL-60 cells. 3-Dimensional drug interaction analysis was performed for binary combinations of CP-4055 in HL-60 cells as described in Methods. The combination effect surface is presented as a color-coded contour map where green represents additivity, red antagonism and blue synergy. For clarity, the color spectrum has been constrained to the range −6 to +6. Values beyond those extremes were observed, but not discriminated (i.e. the blue corresponding to CE = 6 actually represents CE ≥ 6).

**Figure 6:**
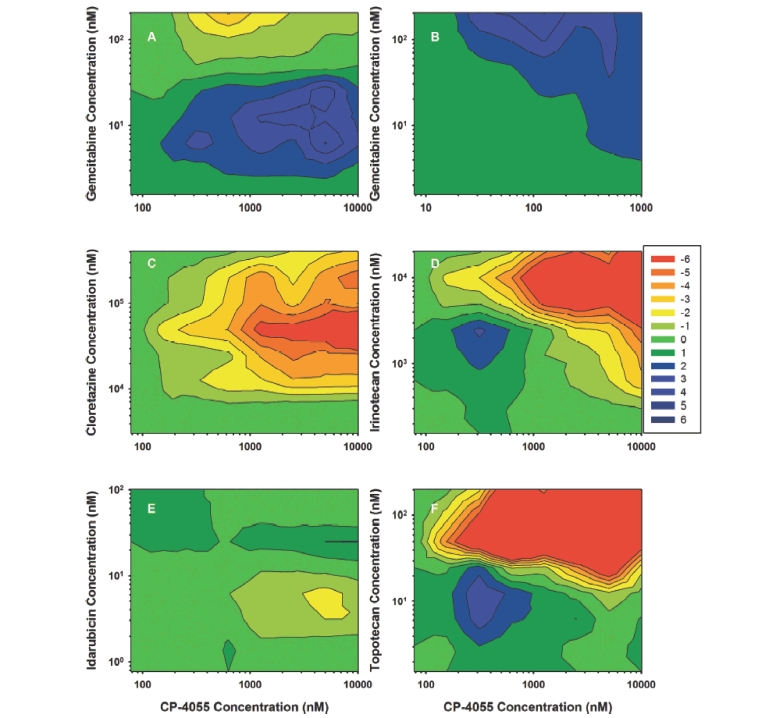
Effect of ratio on drug interaction in U937 cells. 3-Dimensional analysis was performed as in Figure 5 for the same drug combinations but in the U937 cell line.

In HL-60 cells, the CP-4055 + gemcitabine simultaneous combination is highly synergistic across a wide range of drug ratios as evidenced by the large rectangle of blue contours (Figure 5, panel A). The map for the cytarabine + gemcitabine combination is similar; although the area of high synergy is reduced (panel B). Areas of significant synergy are also observed with the other drug combinations, but the majority of the maps are in the additive (green) range. Notably, there are very few drug ratios in the antagonistic range (orange-red) in HL-60 cells. Results in HL-60 contrast with those seen in U937 cells (Figure 6). The synergistic region of the CP-4055 + gemcitabine map remains predominant, but a small region of moderate antagonism is apparent. Likewise, the cytarabine + gemcitabine map remains additive to synergistic. In contrast, strong antagonism was observed at certain drug ratios in the combinations of CP-4055 with cloretazine (CE = −12 max; panel C), irinotecan (CE = −15 max; panel D) and topotecan (CE = −16 max; panel F). These values tend to occur in the upper right-hand quadrant of the map where the CP-4055:cloretazine ratio is <0.05; CP-4055:irinotecan is <0.5, and CP-4055:topotecan is <10. The interaction of CP-4055 with idarubicin is predominantly additive and ratio independent, which is particularly important since this will be the first combination tested in the clinic. Of note, evaluation of the impact of drug ratio as a function of drug sequence indicates that the CP-4055-idarubicin interaction is both ratio and sequence-independent in both cell lines (data not shown).

### Exposure time impacts individual response to CP-4055 and gemcitabine, but not the drug interaction

**Figure 7:**
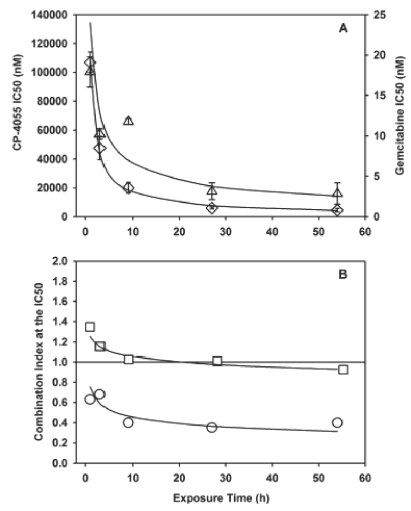
Effect of exposure time on the interaction of CP-4055 plus gemcitabine. Panel A: IC50 versus exposure time in HL-60 cells Is shown for CP-4055 (diamonds) and gemcitablne (triangles) as single agents. Panel B: HL-60 (circles) or U937 (squares) cells were exposed simultaneously to CP-4055 in combination with gemcitabine at a fixed ratio of 1:0.0025 or 1:0.02, respectively. The combination index at the IC50 level of effect was then determined at each indicated time point. Bars: intra-assay standard error; lines: fit of data to the pharmacodynamic model *C^n^* × *T* = *k*.

Traditionally in drug development, more emphasis is placed on the impact of drug concentration on antitumour activity than on the effect of exposure time. However, both are important for activity of agents alone and in combination [27]. Consequently, concentration-time analysis was performed on the synergistic combination of CP-4055 + gemcitabine in HL-60 cells [Figure 7(A)]. Similar results were obtained for U937 cells (data not shown) and revealed that the anti proliferative activity of both CP-4055 and gemcitabine conforms reasonably well to the pharmacodynamic model *C^n^* × *T* = *k*, where *C* is the IC50 concentration, *T* is exposure time, *n* is the concentration coefficient and *k* is an exposure constant (Table IV). The value for *n* indicates the relative impact of concentration versus time to effect. In the case of HL-60 cells, CP-4055 concentration has more impact than time (*n* = 2), whereas in U937 cells the contribution is similar (*n* = 1). The two factors contribute equally for gemcitabine in both cell lines. Interestingly, drug interaction is not markedly affected by exposure time as shown in Figure 7(B). The combination index (at the IC50) drops somewhat from 1 to 9 h and then remains constant to a maximum of 54 h.

**Table IV:** Pharmacodynamic model parameters for the simultaneous combination of CP-4055 plus gemcitabine.*

	HL-60			U937		
		
Drug	*n*	Log(*k*)	*R*^2^	*n*	Log(*k*)	*R*^2^
CP-4055	1.78	9.11	0.86	1.09	4.47	0.84
Gemcitabine	1.23	1.59	0.99	1.06	2.89	0.87

* Based on log(*T*) = n log(*C*) + log(*k*), where *C* is the IC50 concentration, *n* is the concentration coefficient, *k* is the exposure constant and *R*^2^ is the Pearson linear correlation coefficient.

## Discussion

Traditionally, selection of cytotoxic drugs for combination chemotherapy has been empirical based on differing mechanisms of action, non-overlapping toxicities, overcoming multi-drug resistance, and increasing dose-density to exploit tumour growth kinetics. Clinically, agents are combined at their maximum tolerated dose and associated schedule as determined in late stage clinical trials. Even the newer combinations of cytotoxic drugs with targeted therapeutics follow these general principles. Often however, drug combinations are evaluated in the clinic without prior pre-clinical studies to help guide the clinical protocol. The current work therefore assessed the impact of drug sequence of administration, drug ratio and drug concentration versus exposure time on interactions between the lipophilic cytarabine analog, CP-4055 and several important drugs for the treatment of human leukemia and lymphoma.

CP-4055 is a pro-drug that generates cytarabine following serum or intracellular esterase cleavage of the elaidic acid moiety. In vitro anti proliferative activity of CP-4055 compared with cytarabine has been reported previously in other human leukemia models using different assays and exposure times (Table V). Of note, results of these experiments are expressed in terms of CP-4055 and not the cytarabine produced or the active metabolite, ara-CTP. Relative activity of CP-4055 to cytarabine is highly variable across cell lines and even within the same cell line, with CP-4055 to cytarabine IC50 ratios ranging from 0.04 to 9. Unlike these studies, the current work assessed survival of viable cells using an ATP metabolic endpoint, which our laboratory has found to correlate well with the MTS mitochondrial metabolic assay and with the propidium iodide assay for total nucleic acids, but with greater sensitivity. By ATP assay, CP-4055 was active against HL60 leukemia and U937 lymphoma cells with an IC50 that is clinically achievable, since the *C*_max_ for CP-4055 in patients with solid tumours is 64 μM and in hematological malignancies as high as 700 μM [13]. Cytarabine was 4-fold more potent than CP-4055 in both cell lines, suggesting that esterase activation of CP-4055 was less efficient in these models. The 20-fold difference in sensitivity in HL-60 versus U937 to both drugs was notable. The relative resistance of HL-60 cells was not due to lack of immunoreactive deoxycytidine kinase in this cell line [28], however other metabolic factors could be involved, including 5′-nucleotidase, cytidine deaminase and DNA polymerase [2].

**Table V:** Reported potency of CP-4055 versus cytarabine.

	IC50 (nM)					
					
Cell line	Cytarabine	CP-4055	Ratio^1^	Assay	N	Reference
CCRF-CEM	50	ND^4^		SRB^2^	119	NCI^3^
CCRF-CEM	40	70	1.75	Cell Count	3 – 4	Breistol (ref. 10)
HL-60(TB)	200	ND^4^		SRB	119	NCI
K-562	15849	1140	0.07	SRB	119	NCI
RPMI-8226	316228	172330	0.54	SRB	119	NCI
SR	251	973	3.87	SRB	119	NCI
MOLT-4	50	2	0.04	SRB	119	NCI
MOLT-4	0.1	0.9	9	Cell Count	3 – 4	Breistol (ref. 10)
U937	167	710	4.25	ATP	14	Current study
HL-60	2989	13387	4.48	ATP	24	Current study

1 Ratio of the IC50 for CP-4055 to that of cytarabine.

2 Sulforhodamine B stain for total protein.

3 National Cancer Institute 60 cell line screen (http:dtp.nci.nih.gov; cytarabine is NSC 63878).

4 Not determined.

Gandhi and colleagues [29,30] were the first to describe biochemical modulation of cytarabine by drugs like fludarabine that inhibit ribonucleotide reductase and thereby increase the rate of ara-CTP accumulation in leukemia. Clinical activity of the combination of cytarabine with fludarabine has been documented in AML and in refractory or relapsed ALL with further enhancement of efficacy by addition of G-CSF to create the so called FLAG regimen [4]. Drug synergy has been observed subsequently for the combination of cytarabine with gemcitabine in HL-60 cells [ratio 1:0.44; CI = 0.82 [6]], as well as in AML patient samples [CI = 0.5 [5]]. The current work found comparable synergistic activity in HL-60 cells with the CP-4055-gemcitabine combination (CI = 0.4). Moreover, this interaction was largely independent of drug sequence, drug ratio, and exposure time, which is advantageous for clinical translation. Since the CP-4055-gemcitabine interaction could exploit the topoisomerase I mechanism of gemcitabine [24], interaction with other topoisomerase I inhibitors was investigated. Moderate to strong synergy was observed with CP-4055 and the topoisomerase I inhibitors irinotecan and topotecan, but only when administered after CP-4055. Administration of these camptothecin analogs prior to CP-4055 resulted in additive to antagonistic interactions.

Drug ratio analysis demonstrated the importance of this parameter in guiding clinical translation. While the CP-4055-gemcitabine map indicated a broad range of synergistic ratios, maps for combinations of CP-4055 with the camptothecins and cloretazine indicated that strong antagonistic interactions could occur if drug ratio was not carefully controlled. Regulation of fixed drug ratios is feasible in vitro, but problematic in vivo. Mayer et al. [31] have recently confirmed the need to control ratios of antitumor drugs, including the combination of cytarabine with the anthracycline daunorubicin. More important, they have demonstrated that in vivo control is now possible by encapsulating the two agents at their synergistic ratio in liposomal carriers. Pre-clinical evaluation of drug interaction coupled with this technology could greatly improve antitumor activity. In addition, this approach could shift design of combination regimens from the current model, which assumes that maximum therapeutic activity requires maximum dose intensity for all drugs in the combination, to one in which the subtleties of drug interaction are recognised. The CP-4055-gemcitabine combination is a prime example, where optimal synergy occurs at CP-4055 to gemcitabine molar ratios of 400 to 1. Therefore, even as targeted therapeutics take center stage in anticancer drug development, there remains much room for optimizing the use of traditional cytotoxic drugs to improve treatment of human cancer.
